# Effects of *Panax ginseng*, *Turnera diffusa* and *Heteropterys tomentosa* extracts on hippocampal apoptosis of aged rats

**DOI:** 10.1590/S1679-45082013000200005

**Published:** 2013

**Authors:** Andréia Gomes Bezerra, Soraya Soubhi Smaili, Guiomar Silva Lopes, Elisaldo Araújo Carlini

**Affiliations:** 1 Universidade Federal de São Paulo, São Paulo, SP, Brazil

**Keywords:** Plants, medicinal, Apoptosis, Aging, Hippocampus

## Abstract

**Objective:**

To verify if the medicinal plants *Panax ginseng C.A. Mey, Turnera diffusa Willd. ex Schult*., and *Heteropterys tomentosa O. Mach*., which are amply used by the population as tonics and cognition enhancers, could have a protective effect on cell death by apoptosis, since this could be one of the mechanisms of action of these substances.

**Methods:**

Aged male Wistar rats (n=24) were divided into four groups. Over 30 days, three groups received treatments with hydroalcoholic extracts of the plants, and one group received saline solution. A fifth group with young adult male Wistar rats (n=4) received saline solution during the same period. Using the TUNEL technique, the percentage of apoptosis in the hippocampus of these animals was evaluated.

**Results:**

No differences were observed between the percentage of apoptotic cells in the hippocampus of aged animals and of young control animals. The percentage of apoptosis in the hippocampus of aged animals treated chronically with the extracts from the three plants also did not differ from the percentage of apoptosis in the hippocampus of the control group of aged animals.

**Conclusion:**

Treatment with the hydroalcoholic extracts of *Panax ginseng, Turnera diffusa,* and *Heteropterys tomentosa* did not influence the apoptosis of the hippocampal cells of aged rats.

## RESUMO

**Objetivo:**

Plantas medicinais, como *Panax ginseng C.A. Mey, Turnera diffusa Willd. Ex Schult*. e *Heteropterys tomentosa O. Mach*. são amplamente utilizadas pela população como tônicas e para melhora da cognição. O presente estudo verificou se essas plantas poderiam ter algum efeito protetor na morte celular por apoptose, podendo este ser um dos mecanismos de ação dessas substâncias.

**Métodos:**

Ratos machos Wistar idosos (n = 24) foram divididos em quatro grupos. Durante 30 dias, três grupos receberam tratamento com extratos hidroalcoólicos das plantas e um grupo recebeu solução salina. Um quinto grupo com ratos machos Wistar adultos jovens (n = 4) recebeu solução salina durante o mesmo período. Utilizando-se a técnica de TUNEL, avaliou-se a porcentagem de apoptose no hipocampo desses animais.

**Resultados:**

Não foram observadas diferenças entre a porcentagem de células apoptóticas no hipocampo de animais idosos e de animais jovens controles. A porcentagem de apoptose no hipocampo dos animais idosos tratados cronicamente com os extratos das três plantas também não diferiu da porcentagem de apoptose do hipocampo dos animais idosos do grupo controle.

**Conclusão:**

O tratamento com os extratos hidroalcoólicos de *Panax ginseng, Turnera diffusa* e *Heteropterys tomentosa* não influenciou a apoptose das células hipocampais de ratos idosos.

## INTRODUCTION

The process of aging causes a series of physiological and anatomical alterations^(1)^. Among these changes, the gradual loss of various cognitive functions is worth mentioning, including attention, reasoning, orientation, language, and especially memory^(2)^. In this case, the hippocampus, a brain structure intimately related to memory, would be particularly compromised^(3)^.

It is known that neuronal loss is a process that does not occur on a large scale or homogeneously in all areas of the brain, but it is suggested that it is involved in cognitive decline resulting from aging^(4)^. Among the biological mechanisms of neuronal loss, apoptosis stands out and can be related to diverse pathogenic processes. An excess in apoptosis rates is related to cellular degeneration caused by oxidative stress^(5,6)^, frequently associated with aging^(7,8)^. Apoptosis is also related to pathogenesis of neurodegenerative conditions, such as Alzheimer's disease^(9)^, Huntington's disease^(10)^ or Parkinsonism^(11)^. Apoptosis may also be involved in the deficit of memory and in hippocampal function^(12)^.

The plants studied in this paper, *Panax ginseng, Turnera diffusa* (popularly known as *“damiana”),* and *Heteropterys tomentosa* (popularly known as *“nó-decachorro”*, in Portuguese), have widespread use that coincides with a possible nootropic or adaptogenic action. Nootropic are drugs characterized by improvement of a person's cognitive performance, and do not possess a specific chemical class or mechanism of action^(13)^. Adaptogenic substances, on the other hand, promote increased physical performance or resistance to stress^(14)^.

*Panax ginseng* was chosen since there are several studies in literature that confirm its adaptogenic action and show positive effects on memory, evaluated by means of improved performance of rats submitted to avoidance testing^(15)^. It was also observed that ginseng extract has a protective effect on death by apoptosis in SK-N-MC neuron cells^(16)^ and astrocytes^(17)^.

A bibliographic survey points out that *Turnera diffusa* and *Heteropterys tomentosa* have uses that fit in with the adaptogenic concept and are among those most often used by the Brazilian population^(18)^, but there are not many scientific studies confirming these beneficial effects. *Turnera diffusa* is described as a stimulant, tonic, and aphrodisiac agent^(19)^. The possible aphrodisiac action, its primary popular reason for usage, was assessed in a pharmacological investigation^(20)^. In this study, hydroalcoholic fluid extracts of *Turnera diffusa* and *Pfaffia paniculata* improved the copulatory performance of sexually incapacitated rats. In a passive avoidance test, a standardized extract of *Heteropterys tomentosa* was effective in reversing the memory deficit in aged animals^(21)^. The plant also showed a good antioxidant index over lipoperoxidation *in vitro* ^(22)^.

## OBJECTIVE

Considering cognitive decline resulting from aging, the importance of the hippocampus for this process and the potential neuroprotective effect of the plants under consideration, this study aimed to evaluate the effects of hydroalcoholic extracts (HE) of the roots of *Panax ginseng*, of the aerial portions of *Turnera diffusa,* and of the roots of *Heteropterys tomentosa* on the apoptosis rate of hippocampal cells in aged rats.

## METHODS

### Botanical material

Aerial parts of *Turnera diffusa* were obtained commercially, from Quimer Ltda. (batch 011, valid until November, 2006), while the roots of *Panax ginseng* (batch GINP04/02, valid until June, 2008) and *Heteropterys tomentosa* (batch NOC06/02, valid until August, 2008), in powder form, were obtained commercially from Santos Flora^®^, all of them provided with official proof of botanical identification of the species. The HEs were prepared by the turbolysis technique^(23)^. Posteriorly, they were freeze-dried (freeze dryer EDWARDS Pirani 501) to obtain dry extracts, which were stored inside a desiccator in the refrigerator.

### Animals

Twenty-four male Wistar aged rats were used (20 month-old) along with four young adult rats (3 month-old), provided by the experimental animal facility – Department of Psychobiology of the *Universidade Federal de São Paulo* (UNIFESP). All animals were maintained in rooms with controlled temperature (23±2°C) and a 12-hour light/dark cycle, with water and food *ad libitum*. The animals were euthanized by decapitation. The project was submitted to and approved by the Ethics Committee of UNIFESP (protocol number 0464/05).

### Treatment of the animals

The aged rats were divided into four groups and received the treatments orally for 30 days. The groups received 100mg/kg of *Panax ginseng* extract, a dosage effective in learning and memory of rats^(15)^, 50mg/kg of *Heteropterys tomentosa*, a dosage that reversed the memory impairment of aged rats^(21)^, and 500mg/kg of *Turnera diffusa*. Both control groups (aged control group and young control group) received saline solution orally for 30 days.

On the 30^th^ day of treatment, one hour after administration, the animals were anesthetized with ketamine (0.8mL/kg) and xylazine (0.5mL/kg) and submitted to transcardiac perfusion with 4% formaldehyde solution. Next, the animals were decapitated, and the brains were carefully removed and stored in 4% formaldehyde for 48 hours for further paraffin inclusion.

### Analysis of apoptosis by the TUNEL (TdT-mediated dUTP-biotin nick end-labeling) immunohistochemical method

To use the TUNEL method, a kit was acquired (ApopTag Plus-peroxidase from ONCOR, Inc., USA), following the manufacturer's protocol and the description by Lopes et al.^(8)^.

The animal brains included in paraffin were sliced with a microtome. Tissue sections were deparaffinized with 95 to 70% ethanol and washed in PBS. After the immunohistochemical reaction sequence, the color of positive cells was revealed with the use of diaminobenzidine (DAB) and the use of Carrazi hematoxylin for counterstaining.

The slides were observed under a light microscope. The percentage of positive cells was calculated for the TUNEL reaction in the hippocampus regions (CA1, CA3, and dorsal and ventral dentate gyri). The percentage was obtained by counting the number of cells marked by the reaction relative to the total number of cells present in the granular layers of the said regions [(apoptotic cells/total number of cells) X 100].

### Statistical analysis

For data analysis, one-way ANOVA was used. The results were expressed as mean±standard error of the mean (SEM) and a 5% significance level was adopted (p<0.05).

## RESULTS

On table 1, we observe that the percentage of death in the hippocampal regions analyzed by the TUNEL reaction showed no statistically significant differences between the groups of aged and young animals. Additionally, the aged rats treated with the three different plant extracts did not differ from the animals with no treatment.

**Table 1:** Percentage of positive-TUNEL cells in different hippocampal regions [CA1, CA3, dorsal dentate gyrus and ventral dentate gyrus] of three groups of aged animals treated with hydroalcoholic extracts of *Panax ginseng*, hydroalcoholic extracts of *Heteropterys tomentosa* and hydroalcoholic extracts of *Turnera diffusa*, besides controls, with no treatment. The results represent mean±SEM. ANOVA. No statistically significant results were found

Group/treatment (n)	% of positive-TUNEL cells			
	CA1	CA3	DDG	VDG
Aged CTL (7)	0.03±0.03	0±0	0.02±0.02	0±0
Young CTL (4)	0±0	0.11±0.11	0.05±0.03	0.06±0.06
Aged PG (6)	0.10±0.06	0.05±0.05	0±0	0.03±0.03
Aged HT (6)	0.07±0.04	0.12±0.08	0±0	0±0
Aged TD (5)	0.10±0.07	0.07±0.07	0±0	0.03±0.03

DDG: Dorsal dentate gyrus; VDG: Ventral dentate gyrus; PG: *Panax ginseng;* HT: *Heteropterys tomentosa;* TD: *Turnera diffusa;* CTL: Control.

## DISCUSSION

This present study aimed to assess the possible anti-apoptotic effect of three HEs (*Panax ginseng*, *Turnera diffusa*, and *Heteropterys tomentosa*) during aging. The animals were treated for 30 days with the plant HEs under study. The brains of these animals were removed for posterior TUNEL reaction. Regions CA1, CA3, and dorsal and ventral dentate gyri of the hippocampus were analyzed as to percentage of neuron death. The regions analyzed by the TUNEL reaction showed no statistically significant differences among the groups of aged animals treated with the three different plant extracts.

There are various studies of vegetable-origin substances aiming at an anti-apoptotic effect on the central nervous system cells^(24–26)^. Methanolic extracts of *Acori graminei* and *Uncariae ramulus et uncus* protected the hippocampus from cellular death induced by ischemia^(24)^. A Chinese medicine preparation called Toki-shakuyaku-san showed a neuroprotective effect against the beta-amyloid protein, an effect attributed to its antioxidant action^(26)^. The injection of *Carthamus tinctorius* extract in rats decreased the area of cerebral infarct and the expression of caspase-3 (a pro-apoptotic protein)^(25)^. These studies prove that extracts from vegetable species can show a protective effect against neuron death in various animal models. However, despite the plausibility of the statement above, in the present study, no protection was noted against neuron death by apoptosis.

Additionally, in this study, there were no differences between the percentages of apoptotic cells in the group of aged animals and the group of young animals, both without treatment. This fact raises questions as to the statement that aging and neuron loss are concomitant events. In fact, neuron loss in aging is discussed by many authors. Part of them has demonstrated that aging is associated with a physiological decrease in the total number of cells in some regions of the brain. In this sense, Ishimaru et al.^(27)^ observed that the number of cells in the hippocampus of aged rats is 10 to 30% lower than in young rats. An increase in apoptosis was found in the striate of adult and aged rats by means of the TUNEL reaction, which could be related to this cellular decrease in the central nervous system^(28)^. However, other studies suggested that this neuron loss during aging is smaller than in newborns, when comparing the brains of newborn rats with those of adult and aged rats^(29)^. These authors noted that in the neocortex, brainstem, hippocampus, and cerebellum, there was a smaller number of apoptotic cells in aged than in newborn animals. In the newborns, this high rate of apoptosis is intimately connected with development, proliferation, and cellular migration^(29)^. Other authors also analyzed the bulb, cerebellum, and corpus callosum in aged animals^(30)^. Thus, the relation between aging and apoptosis is still not totally defined.

The data of the present study are in accordance with some findings in literature that describe the absence of a large number of apoptotic cells in the brain of aged animals. The results of the present study also suggest that the medicinal plants evaluated do not have an anti-apoptotic effect on the central nervous system. Nevertheless, for the correct interpretation of the data, some methodological reservations and limitations must be considered. First, given that there were no high apoptosis rates in the aged animals, any anti-apoptotic effects of the plants analyzed could have been concealed. In this respect, in future studies it would be useful to use methods of induction of apoptosis and neuron death. Furthermore, only neuron death by apoptosis was addressed, omitting mechanisms such as necrosis and autophagy. In this way, one cannot conclude that these plants do not possess a protective effect against neuron death in general. Finally, one should consider that apoptosis induced by aging is not restricted to the hippocampus. Thus, studies that investigate the effects of the plants analyzed on other brain structures may be of great worth.

It is important to remember that the plants studied are commonly used by the general population that recognizes its effects as adaptogens or nootropics. Scientific studies with vegetable substances that display neuroprotection and anti-apoptotic effects are scarce when compared to the quantity of plants described with this finality, whether popular or with scientific literature data. In this respect, it is important to deepen these studies in order to clarify the mechanism of action of these plants, so widely used by the population.

## CONCLUSION

The extracts of *Panax ginseng, Turnera diffusa,* and *Heteropterys tomentosa* do not display any effect on apoptosis rates of the hippocampus of aged rats. These results are specific for the experimental conditions used, and it is necessary to evaluate the effect of these plants by means of studies that induce apoptosis or that assess cognitive decline typical of aging by means of other neurobiological mechanisms.

## References

[1] Knox CA, Albert ML, Koefel JE (1994). Neuroanatomical changes associated with aging in the peripheral nervous system. Clinical neurology of aging.

[2] Albert MS, Albert ML, Koefel JE (1994). Age-related changes in cognitive function. Clinical neurology of aging.

[3] Lister JP, Barnes CA (2009). Neurobiological changes in the hippocampus during normative aging. Arch Neurol.

[4] Shankar SK (2010). Biology of aging brain. Indian J Pathol Microbiol.

[5] Whittemore ER, Loo DT, Cotman CW (1994). Exposure to hydrogen peroxide induces cell death via apoptosis in cultured rat cortical neurons. Neuroreport.

[6] Chandra J, Samali A, Orrenius S (2000). Triggering and modulation of apoptosis by oxidative stress. Free Radic Biol Med.

[7] Floyd RA (1991). Oxidative damage to behavior during aging. Science.

[8] Lopes GS, Mora OA, Cerri P, Faria FP, Jurkiewicz NH, Jurkiewicz A (2004). Mitochondrial alterations and apoptosis in smooth muscle from aged rats. Biochim Biophys Acta.

[9] Mattson MP (2004). Pathways towards and away from Alzheimer's disease. Nature.

[10] Rosenstock TR, Carvalho AC, Jurkiewicz A, Frussa-Filho R, Smaili SS (2004). Mitochondrial calcium, oxidative stress and apoptosis in a neurodegenerative disease model induced by 3-nitropropionic acid. J Neurochem.

[11] Ziv I, Melamed E, Nardi N, Luria D, Achiron A, Offen D (1994). Dopamine induces apoptosis-like cell death in cultured chick sympathetic neurons – a possible novel pathogenetic mechanism in Parkinson's disease. Neurosci Lett.

[12] Zarifkar A, Choopani S, Ghasemi R, Naghdi N, Maghsoudi AH, Maghsoudi N (2010). Agmatine prevents LPS-induced spatial memory impairment and hippocampal apoptosis. Eur J Pharmacol.

[13] Benesová O (1994). Neuropathobiology of senile dementia and mechanism of action of nootropic drugs. Drugs Aging.

[14] Wagner H, Nörr H, Winterhoff H (1994). Plant adaptogens. Phytomedicine.

[15] Petkov VD, Mosharrof AH (1987). Age- and individual-related specificities in the effects of standardized ginseng extract on learning and memory (experiments on rats). Phytother Res.

[16] Lee J, Kim J, Cho S, Kim Y, Choi K, Joo W (2004). Protective effect of ginseng extract against apoptotic cell death induced by 2,2′, 5,5′ tetrachlorobiphenyl in neuronal SK-N-MC cells. Life Sci.

[17] Naval MV, Gómez-Serranillos ME, Carretero AMV (2007). Neuroprotective effect of a ginseng (Panax ginseng) root extract on astrocytes primary culture. J Ethnopharmacol.

[18] Mendes FR, Carlini EA (2007). Brazilian plants as possible adaptogens: an ethnopharmacological survey of books edited in Brazil. J Ethnopharmacol.

[19] Mors WB, Rizzini CT, Pereira NA, DeFilipps RA (2000). Medicinal plants of Brazil.

[20] Arletti R, Benelli A, Cavazzuti E, Scarpetta G, Bertolini A (1999). Stimulating property of Turnera diffusa and Pfaffia paniculata extracts on the sexual behavior of male rats. Psychopharmacology.

[21] Galvão SMP, Marques LC, Oliveira MGM, Carlini EA (2002). Heteropterys tomentosa (extract BST0298): a Brazilian plant that improves memory in aged rats. J Ethnopharmacol.

[22] Mattei R, Barros MP, Galvão SMP, Bechara EJH, Carlini EA (2001). Heteropteris tomentosa O. Machado: effects of extract BST 0298 on the oxidative stress of young and old rat brains. Phytother Res.

[23] Franco SL (1990). Maytenus ilicifolia martius ex. Reiss. Celestraceae – Proposta tecnológica de macerados [tese].

[24] Lee B, Choi Y, Kim H, Kim SY, Hahm DH, Lee HJ (2003). Protective effects of methanol extract of Acori graminei rhizome and Uncariae ramulus et uncus on ischemia-induced neuronal death and cognitive impairments in the rat. Life Sci.

[25] Luo J, Fang ZP, Zhou LM, Lai ST (2004). [Effects of Carthamus tinctorius injection on bcl-2, caspase-3 expression related to neurons apoptosis after local cerebral ischemia]. Zhongguo Zhong Yao Za Zhi.

[26] Egashira N, Iwasaki K, Akiyoshi Y, Takagaki Y, Hatip-Al-Khatib I, Mishima K (2005). Protective effect of Toki-shakuyaku-san on amyloid beta 25-35-induced neuronal damage in cultured rat cortical neurons. Phytother Res.

[27] Ishimaru H, Ogawa S, Fuji K, Fukuta T, Kaneyama T, Nabeshima T (1991). Aged-related changes in learning and memory, choline acetyltransferase activity and number of neuronal cells in rats. J Pharmacobio-Dyn.

[28] Zhang L, Kokkonen G, Roth GS (1995). Identification of neuronal programmed cell death in situ in the striatum of normal adult rat brain and its relationship to neuronal death during aging. Brain Res.

[29] White LD, Barone S (2001). Qualitative and quantitative estimates of apoptosis from birth to senescence in the rat brain. Cell Death Differ.

[30] Dorszewska J, Adamczewska-Goncerzewicz Z, Szczech J (2004). Apoptotic proteins in the course of aging of central nervous system in the rat. Respir Physiol Neurobiol.

